# Hyperkinetic Biliary Dyskinesia: An Underrecognized Problem With Good Surgical Outcomes After Cholecystectomy

**DOI:** 10.7759/cureus.63237

**Published:** 2024-06-26

**Authors:** Kelsi D Camacho, Ryan B Cohen, Sonam Kapadia, Neha Gondra, Josue D Parr, Mason J Kaneski, Hazem Shamseddeen, Jonathan L Pierce, Hung S Ho, Shushmita M Ahmed, Mohamed R Ali, Victoria Lyo

**Affiliations:** 1 Surgery, University of California Davis School of Medicine, Sacramento, USA; 2 Surgery, University of California Davis Medical Center, Sacramento, USA; 3 Surgery, Los Angeles County Department of Health Services, Los Angeles, USA; 4 Center for Alimentary and Metabolic Sciences, University of California Davis School of Medicine, Sacramento, USA; 5 General Surgery, University of California Davis School of Medicine, Sacramento, USA

**Keywords:** cholelithiasis, cholecystectomy, biliary dyskinesia, hyperkinetic gallbladder, hyperkinetic biliary disease

## Abstract

Introduction

While surgical indications for symptomatic cholelithiasis and biliary hypokinesia are clear, hyperkinetic biliary dyskinesia (HBD) is an underrecognized condition with poorly defined symptomology and management guidelines. HBD is typically defined as a gallbladder ejection fraction (EF) ≥ 80% on a hepatobiliary iminodiacetic acid (HIDA) scan. We aimed to identify the prevalence and radiographic reporting of HBD, physician referral patterns, and clinical outcomes following cholecystectomy.

Methods

A retrospective cohort study of patients with HIDA scans completed over 21 years at our tertiary care hospital was performed. Demographics, symptomatology, referral patterns, and operative data were collected. HBD was defined as HIDA EF *≥*80%. Patients with HBD who underwent cholecystectomy were analyzed. ANOVA and chi-square tests were used to compare variables among patients with or without symptom improvement using Statistical Product and Service Solutions (SPSS; IBM SPSS Statistics for Windows, Armonk, NY).

Results

Of 1,997 patients (73% female, mean age 51.7 years) who had HIDA scans with reported EF, 730 (36.6%) had an EF≥80%. Only 13.7% of HIDA scans with EF≥80% were reported as hyperkinetic, and the rest are “normal”. Cholecystectomy was performed in 57 (7.8%) patients with EF≥80%, most being elective (89.5%) and all minimally invasive. Primary care physicians* (*PCPs) referred most elective cases to surgery (61.4%). The median time from HIDA to cholecystectomy was 146 days. Chronic cholecystitis was common in pathology (82.5%), while 38.6% had cholelithiasis. Overall, 53 patients (93.0%) reported symptom improvement at a median follow-up of 17.0 days. Patients without improvement had a higher prevalence of chronic gastrointestinal conditions (p<0.05), but not significantly more cholelithiasis, cholecystitis, time to surgery, or elective surgery status.

Conclusions

HBD is common but often underdiagnosed and thus likely underrecognized by treating physicians. Most HBD patients benefit from cholecystectomy, regardless of cholelithiasis. Patients with persistent symptoms after cholecystectomy may have confounding gastrointestinal diagnoses. Increased awareness among radiologists, referring PCPs, gastroenterologists, and surgeons about HBD and postoperative outcomes is needed to ensure that HBD is adequately treated.

## Introduction

Laparoscopic cholecystectomy for symptomatic cholelithiasis is the most common elective operation performed in the U.S. and is widely understood by primary care, emergency physicians, and surgeons [1]. While the surgical indications for symptomatic cholelithiasis and biliary dyskinesia are well-defined, based on substantiative data [1,2], hyperkinetic biliary dyskinesia (HBD) is a unique and underrecognized phenomenon that does not fall into the classic biliary management paradigms. First described in 1999 as biliary hyperkinesia or hyperkinetic gallbladder, the phenomenon is defined as a gallbladder ejection fraction (EF)≥80% on a hepatobiliary iminodiacetic acid (HIDA) scan [3]. Over the last several decades, the surgical management of HBD has been validated by a growing body of evidence showing effective symptom improvement after cholecystectomy ranging from 76-100% [3-15]. A recent meta-analysis of 332 patients reported 91.3% symptomatic improvement after cholecystectomy [14].

Nonetheless, HBD remains a little-known phenomenon without general acceptance in the general surgery or medical lexicon. Under the ROME IV criteria, there is no specific determination for HBD as a functional gallbladder disorder, including whether the presence of cholelithiasis should exclude the diagnosis [16]. Furthermore, the definitions used for HBD and whether to include patients with cholelithiasis are variable in the literature [5-7,9-15]. Moreover, there is a paucity of data about whether specific patient populations or other preoperative characteristics may respond better to surgical management.

We hypothesize that HBD is an underrecognized phenomenon due to a lack of awareness among surgeons, medical specialists, and primary care providers (PCP). The purpose of our study was to identify the prevalence and radiographic reporting of HBD at our major, tertiary care center. Additionally, we sought to identify physician referral patterns and clinical outcomes following cholecystectomy for HBD patients who underwent cholecystectomy to identify specific risk factors for non-response to surgical treatment. We hope to shed light on factors limiting the recognition and treatment of HBD. Increased awareness among prescribing physicians and surgeons about HBD and the postoperative outcomes may lead to wider acceptance of the disease process and improved patient outcomes.

This article was previously presented as a meeting abstract at the Society of American Gastrointestinal and Endoscopic Surgeons 2024 Annual Meeting on April 18, 2024.

## Materials and methods

A retrospective cohort study following the Strengthening the Reporting of Observational Studies in Epidemiology (STROBE) guidelines of all adult patients who underwent HIDA scans between 1/1/2002 and 10/01/2023 at our tertiary care hospital was performed after institutional review board approval. The radiological impression and reported EF of each HIDA scan were collected to determine what proportion of HBD cases were interpreted as “normal” versus “hyperkinetic”.

For our cohort, we defined HBD as a HIDA EF≥80% and identified patients who underwent cholecystectomy at our institution. Patients with both HIDA≥80% and who underwent cholecystectomy at our institution were included. Patients with gallstone disease on pre-operative ultrasonography (US), computed tomography (CT) scan, or magnetic resonance imaging were also included in the study if there was no evidence of acute inflammation. Exclusion criteria were EF<80%, no cholecystectomy, and acute inflammation on imaging. Patient demographics, symptomatology, referral patterns, operative data, and post-operative pathology were collected for the cohort, and a chart review was performed by authors (R.C, K.C, S.K., N.G., and J.D.P.) with a review of complex charts by multiple authors and senior author (V.L.). We also compared referring practices by specialty for HIDA scans and surgical referrals to determine if practices varied by specialty.

Symptom improvement was defined as a significant decrease in each patient’s predominant pre-operative symptoms based on postoperative clinic notes at the last follow-up available. Symptoms included right upper quadrant (RUQ) pain, epigastric pain, nausea, and diarrhea. Univariate analysis was used to compare the improvement of symptoms among different demographic, patient-specific, and surgical variables. ANOVA and chi-square tests were used to compare continuous and categorical variables, respectively, between study groups, with a p-value of <0.05 indicating statistical significance performed by one author (R.C.) using the Statistical Product and Service Solutions (SPSS) (IBM SPSS Statistics for Windows, Armonk, NY ) and confirmed by another author (V.L.).

## Results

**Figure 1:**
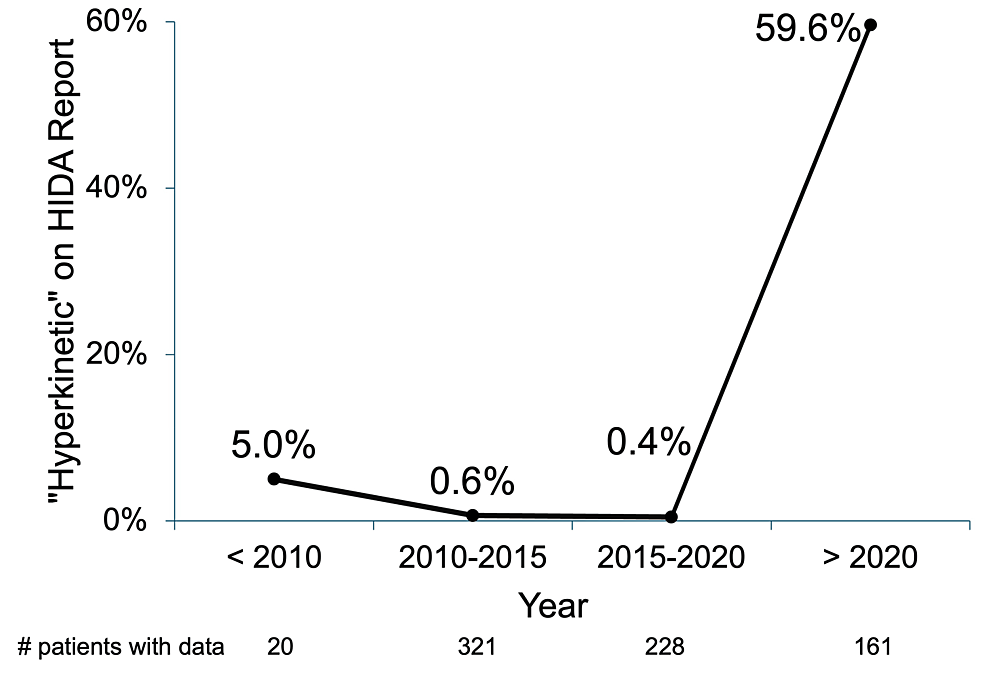
Radiologists' Impressions of HIDA Scans by Timeframe Distribution of HIDA scans with EF≥80% read as “hyperkinetic”. Chi-square analysis demonstrates significantly more studies named “hyperkinetic” from 2020 onward (96/161, 59.6% versus 4/569, 0.7%; p<0.001; chi-square: 368.6). Abbreviation: HIDA, hepatobiliary iminodiacetic acid

Over 21 years, 3,327 total HIDA scans were complete, of which 1,997 (60.0%) reported EF and were performed (73.0% female, with a mean age of 51.715.8 years). Of the 1,997 with reported EF, 730 (36.6%) had EF≥80%, and only 100 (13.7%) were named “hyperdynamic” by radiologists. Interestingly, HIDA scans performed after 2020 compared to those done before with EF≥80% were more likely to be named “hyperdynamic” or “abnormal” (96/161, 59.6% versus 4/569, 0.7%; p<0.001; Figure 1).

Our cohort included 57 patients who had HIDA scans with ≥80% who underwent cholecystectomy. The mean age was 53.6±13.0 years at HIDA scans and 55.4±13.2 years at surgery, with 84.2% of patients being female. At the time of surgical evaluation, the most common symptoms were right upper quadrant/epigastric abdominal pain (50, 87.7%) and nausea (24, 42.1%), while many patients (22, 38.6%) had tenderness on exams, suggestive of inflammation. Four patients had more than one HIDA scan pre-operatively: two had multiple HIDA scans with EF≥80% for which the earliest HIDA scan was used, while the other two patients with duplicate scans had non-reportable EFs.

The majority of HIDA scans were ordered by PCPs (23, 40.4%). Surgery (19, 32.1%) and gastroenterology (16, 28.1%) also ordered HIDA scans. Surgical referrals were most often initiated by PCPs (35, 61.4%) and gastroenterologists (15, 26.3%) with only a minority of referrals coming from other specialists (7,12.3%). When the HIDA scan was read as “normal” by radiology, PCPs appeared more likely than specialists (66.7 vs 33.3%) to refer patients for surgical management. However, there was no statistically significant association between radiographic impression and provider-type ordering surgery referral (Table 1). While most patients (31, 54.4%) were evaluated by gastroenterology prior to referral to surgery, fewer patients (20, 35.1%) had HIDA scans or surgery referral ordered by gastroenterology. Notably, when HIDA impression was “hyperkinetic”, patients were more likely to be evaluated by gastroenterology (18/21, 85.7%) than when read as “normal” (13/36, 36.1%, p<0.001). The median time from HIDA to cholecystectomy was 146 days (interquartile range (IQR): 368) (Table 1). There was no significant difference in the median time to surgery by referring specialty (PCP: 184 (IQR: 350), GI: 111 (IQR: 154), and other specialty (i.e., emergency medicine, bariatric surgery, vascular surgery, general medicine) (41 (1344) days; p=0.327, ANOVA F test: 1.140). Most cholecystectomies were performed electively (89.5%), and all were performed by minimally invasive techniques. Two patients had postoperative complications, including one bile leak and one surgical site infection.

**Table 1:** Referral Patterns for Studies and Surgery Abbreviations: HIDA, hepatobiliary iminodiacetic acid; PCP, primary care physician; GI, gastroenterology; IQR, interquartile range; ᵃ: gastroenterology, emergency medicine, bariatric surgery, vascular surgery, general medicine Chi-square testing was used to compare categorical variables of whether the HIDA impression results were associated with the gastroenterology and surgery referrals. * p<0.001 by chi-square testing

Referral by HIDA Impression	N (%)	P- value	Chi-square
Gastroenterology referral, by HIDA impression			
Normal	36 (63.2%)	<0.001*	13.155
Seen by GI	13 (36.1%)		
Not seen by GI	23 (64.0%)		
Hyperdynamic	21 (36.8%)		
Seen by GI	18 (85.7%)		
Not seen by GI	3 (14.3%)		
Surgery referral, by HIDA impression			
Normal	36 (63.2%)	0.285	1.142
PCP	24 (66.7%)		
Specialist^a^	12 (33.3%)		
Hyperdynamic	21 (36.8%)		
PCP	11 (52.3%)		
Specialist^a^	10 (47.6%)		

Preoperative imaging was performed on all patients (96.5% US) and identified cholelithiasis in 20 (35.1%) patients (Table 2). Cholecystitis (87.7%), more specifically chronic cholecystitis, was the most likely finding on the final pathologic diagnosis (Table 2). Of the 47 patients with chronic cholecystitis, 40.4% had concomitant cholelithiasis on pathology. Of the seven patients with no cholecystitis, 16.7% had concomitant cholelithiasis on pathology. There was some discordance between cholelithiasis on pre-operative imaging (20, 35.1%) and cholelithiasis reported on final pathology (22, 38.6%) (Table 2).

**Table 2:** Preoperative Imaging and Pathologic Details N specifies the total number of each subheading. Abbreviations: US: ultrasound, CT: computed tomography, MRI: magnetic resonance imaging

Imaging Results	N (%)
Pre-op Imaging Performed	57 (100%)
US	55 (96.5%)
CT	1 (1.8%)
MRI	1 (1.8%)
Imaging Findings	
Cholelithiasis	20 (35.7%)
Sonographic Murphy’s sign	3 (5.4%)
Pathologic Results	
Cholelithiasis on pathology	22 (39.3%)
Cholelithiasis on pre-op imaging	17 (77.3%)
No cholelithiasis pre-op	5 (22.7%)
No cholelithiasis on pathology	35 (61.4%)
Cholelithiasis on pre-op imaging	3 (8.6%)
No cholelithiasis pre-op	32 (91.4%)
Cholecystitis on Pathology Without Cholelithiasis	50 (89.3%)
Acute	2 (4%)
Chronic	47 (94%)
Gangrenous	1 (2.0%)
No cholecystitis on pathology	7 (12.3%)
Cholecystitis on Pathology with Cholelithiasis	21 (42.0%)
Acute	1 (50.0%)
Chronic	19 (40.4%)
Gangrenous	1 (100%)
No cholecystitis on pathology	1 (16.7%)

Overall, 53 patients (93.0%) reported symptom improvement at a median follow-up of 17.0 days (IQR: 11.50). The four patients without symptom improvement were all seen by gastroenterology before their surgical evaluation, and two continued to be managed by them. Patients without improvement had a significantly higher prevalence of concurrent gastrointestinal conditions (Table 3, p<0.05). Patients with improved symptoms were not significantly more likely to have cholelithiasis on pathology, cholecystitis, prolonged time to surgery, or elective surgery status (Table 3).

**Table 3:** Clinical Improvement of Symptoms Abbreviations: BMI: body mass index; GI: gastrointestinal; GERD: gastroesophageal reflux disease; HIDA: hepatobiliary iminodiacetic acid ^a^ All GI diagnoses: gastroesophageal reflux disease, irritable bowel syndrome, inflammatory bowel disease, peptic ulcer disease, sphincter of Oddi dysfunction, functional dyspepsia, chronic pancreatitis, gastritis, Barrett’s esophagus, gastroparesis; ^b^ GI diagnoses: same as “a” above but without gastroesophageal reflux disease; ^c^: Psychiatric diagnoses: major depression disorder, anxiety, bipolar disorder, post-traumatic stress disorder, generalized anxiety disorder, substance abuse disorder, panic disorder, obsessive compulsive disorder P values: Chi-square tests were used to compare categorical variables between study groups.

Patient Factors	N	Improved, N (%)	P-value	Chi-Square
Age (years)				
> 50	37	33 (89.2)	0.127	2.325
≤ 50	20	20 (100.0%)		
Sex				
Female	48	44 (91.7%)	0.369	0.807
Male	9	9 (100.0%)		
BMI (kg/m^2^)				
> 30	23	21 (91.3%)	0.683	0.166
≤ 30	34	32 (94.1%)		
All GI Diagnoses^a^				
Present	32	28 (87.5%)	0.067	3.361
Absent	25	25 (100.0%)		
GI Diagnoses^b^,no GERD				
Present	18	14 (77.8%)	0.002*	9.321
Absent	39	39 (100.0%)		
GERD				
Present	27	25 (92.6%)	0.913	0.012
Absent	30	28 (93.3%)		
Psychiatric Diagnoses^c^				
Present	31	28 (90.3%)	0.391	0.737
Absent	26	25 (96.2%)		
Prior Bariatric Surgery				
Yes	7	6 (85.7%)	0.422	0.464
No	50	47 (94.0%)		
Surgical or Pathologic Factors	N	Improved N (%)	P-value	
Time from HIDA to surgery				
> 6 months	27	26 (96.3%)	0.353	0.863
≤ 6 months	30	27 (90.0%)		
Surgery Status				
Elective	51	47 (92.2%)	0.477	0.506
Non-elective	6	6 (100.0%)		
Cholelithiasis on Pathology or Imaging				
Present	25	21 (95.5%)	0.431	.621
Absent	32	32 (91.4%)		
Cholelithiasis on Pathology				
Present	22	21 (95.5%)	0.562	0.336
Absent	35	32 (91.4%)		
Cholelithiasis on Imaging				
Present	20	19 (95.0%)	0.661	0.192
Absent	37	34 (91.9%)		
Cholecystitis on Pathology				
Present	50	6 (92.0%)	0.438	0.602
Absent	7	7 (100.0%)		

## Discussion

There is a growing body of evidence supporting the efficacy of cholecystectomy in the setting of HBD. In our study of 57 patients, we found a symptomatic improvement rate of 93.0% consistent with prior studies, including the largest single-center review to date published in 2023 by Whitaker et al., who reported a symptom improvement rate of 92.3% in 91 patients [6]. A meta-analysis and systematic review by Eltyeb et al. reported symptomatic improvement in 91.3% of 332 reviewed patients [14]. These findings have been reproduced by a multitude of smaller cohort studies over the last decade [5,6,8-15]. Additionally, a recent review by Madura et al. reinforced a cutoff of about 80% for the diagnosis of HBD. In their analysis, which utilized a receiver operating curve analysis of patients above an EF of 50%, 81% represented an optimal cutoff for pain resolution.

Despite the growing evidence for cholecystectomy in this patient population, there seems to be little awareness among PCPs, specialists including gastroenterologists, and even surgeons about the benefits of cholecystectomy in the treatment of HBD. The lack of societal guidelines mirrors this sentiment as well. Under the ROME IV criteria, there is no specific determination for HBD as a functional gallbladder disorder [16], including whether the presence of cholelithiasis should exclude the diagnosis. Increased awareness among radiologists, referring PCPs, gastroenterologists, and surgeons about HBD and postoperative outcomes is needed to ensure HBD is adequately treated.

We hypothesize that this gap in knowledge and practice may be reflected in the diagnostic work-up and referral patterns of HBD patients. These issues span multiple specialties including radiology, PCPs, specialists, gastroenterology, and surgery, which may ultimately result in delayed care or missed opportunities for referral to surgery. This study aimed to analyze patterns for HBD patients and factors that may predict favorable surgical outcomes.

A striking finding of our study was that HBD was present in over one-third of HIDA scans (36.7%), but only 13.7% were noted by radiologists to be abnormal or consistent with HBD. We also found an association between the recentness of HIDA scans with the likelihood of being named “hyperdynamic”, with a drastic increase in radiologic diagnoses from 2020 onward. This may reflect rising awareness in radiology of the disease reflected in the recent literature: three studies on post-cholecystectomy outcomes for HBD were performed before 2019 [3-5] compared to 10 from 2019 onward [6-15]. Presumably, HIDA scans were ordered for abdominal pain with a suspicion of biliary causes. The fact that most studies with an EF>80% were deemed “normal” (86.3%) leaves a wide gap in the care of patients who may have clinical HBD, but may not be referred to surgery due to a lack of awareness of the disease, exacerbated by inadequate radiology reporting.

There appears to be a paucity of data on the diagnosis of HBD in the radiologic literature. As surgeons, we must highlight the problem of hyperdynamic gallbladder disease. Specifically, radiologists should include the possibility of HBD in HIDA scan interpretation to allow ordering providers to consider this disease. The median time from HIDA to surgery was quite long in our cohort (146 days), suggesting inefficiency in the work-up and referral of these patients, which may be the lack of awareness of the disease and the lack of radiographic callout of the diagnosis.

Although not statistically significant, we also found that PCPs were most likely to order HIDA scans, but still nearly one-third were ordered by gastroenterology and surgeons as well. As PCPs are at the frontline of biliary disease work-up and management, it is important to raise awareness of HBD so that PCP can order appropriate testing and proceed with timely referrals. Interestingly, we found that when HBD was noted in the HIDA report, patients were more likely to be referred and evaluated by gastroenterology, highlighting that how findings are reported influences referral patterns.

We found concomitant cholelithiasis in a minority of patients with HBD (35.1% preoperatively, 38.6% on pathology). Most studies on HBD eliminated patients with US evidence of cholelithiasis but did not eliminate patients with gallstones on final pathology [7,9-13]. Some studies were more stringent and eliminated patients with ultrasonography or pathologic diagnoses of stones [5,6,14]. Williford et al. included patients with gallstone disease in their analysis and found an 89% symptom improvement rate [15]. We chose to include patients with pre-operatively identified cholelithiasis in the absence of inflammation to better represent the spectrum of disease of HBD, which may have both a functional component with a concurrent mechanical, obstructive stone component. Patients with cholelithiasis in the absence of cholecystitis typically provide a surgical dilemma, with operative management often deferred or delayed. In practical terms, HIDA is often ordered for patients with minimal symptoms and cholelithiasis to guide further decision-making.

In our analysis, we found that the presence of cholelithiasis was not associated with symptom improvement, suggesting that cholecystectomy for HBD itself, regardless of cholelithiasis presence, is effective. Just as patients are not straightforward, there likely is an overlap between gallstone disease and HBD in the spectrum of biliary disease that has both motility and mechanical obstructive etiologies. On a pathophysiologic level, both the mechanical dynamics of hyperdynamic contracture and gallstone disease may contribute to symptomatology. We felt that it was important to include gallstone pathology in our cohort to better describe the realities of overlapping clinical entities. Surely as cholelithiasis presence does not hinder surgical decision-making to offer cholecystectomy for biliary hypokinesia/dyskinesia, similarly, they should not influence the recognition that concomitant biliary processes may be occurring in HBD.

Interestingly the presence of concomitant gastrointestinal diagnoses was a predictor of persistent pain after cholecystectomy. Comorbid conditions have a confounding effect on the work-up of HBD patients, and care should be taken to perform a thorough evaluation and eliminate other causes of abdominal pain that may be associated with gastrointestinal diagnoses prior to cholecystectomy in these patients. Every HBD patient should be considered on a case-by-case basis with attention to medical and surgical comorbidities and appropriate GI consultation. Additionally, if no other etiologies of pain can be found but HBD is noted, cholecystectomy to rule out biliary causes of pain may be indicated and assist gastroenterology with future management of these complex patients.

Limitations of our study include inherent bias in a retrospective review at a single center. Since this was also performed at a tertiary care center, the cohort may also include more complex patients, limiting some of the generalizability of the data. Additionally, we cannot exclude or tease out the degree to which postoperative symptomatic improvement was due to HBD versus symptomatic cholelithiasis in part of our cohort with concomitant diseases other than to point out that cholelithiasis was not a significant factor in improvement rates. The follow-up interval was not long enough to make meaningful conclusions about long-term outcomes. Nonetheless, similar studies also had short follow-up intervals from one to four weeks, reflecting the normal post-operative visit time interval after cholecystectomy and the limitations of many studies [5-7,11]. Additionally, though a detailed chart review of symptoms was performed, no standardized patient-reported outcome or quality-of-life questionnaires were used to assess outcomes.

## Conclusions

Overall, we found that HBD is often under-reported by radiologists when HIDA scans are performed despite EF meeting diagnostic criteria, though this changed after 2020. Patients presenting with biliary-type pain and HIDA scans with EF≥% and once referred to surgery had good resolution of their symptoms, regardless of the presence of gallstones while concomitant gastrointestinal diagnoses confound outcomes. Our results help reinforce that cholecystectomy is a treatment for HBD and bolster the existing surgical literature. Increased awareness amongst radiologists to report HBD findings on HIDA reports and amongst referring doctors such as PCPs, gastroenterologists, and surgeons is needed to prevent delayed patient care. Special consideration should be reserved for patients with concomitant gastrointestinal diagnoses. Overall, it is imperative that, as surgeons, we prioritize awareness of both the work-up and surgical management of patients with HBD.
